# Angiotensin converting enzyme 2 activation improves allergic rhinitis and suppresses Th2 cytokine release

**DOI:** 10.1002/iid3.763

**Published:** 2023-01-18

**Authors:** Xiuying Sun, Yu Xu, Jinhui Zhou

**Affiliations:** ^1^ Department of Otorhinostomology The Affiliated Huaian No. 1 People′s Hospital of Nanjing Medical University Huai′an Jiangsu China

**Keywords:** ACE2, allergic rhinitis, interleukin, lgE, Th2

## Abstract

**Objective:**

Allergic rhinitis (AR) is primarily regulated by type I hypersensitivity, with Th2 and immunoglobulin E (IgE) playing essential roles. This study aimed to determine whether angiotensin converting enzyme (ACE)2 could participate in the regulation of AR.

**Methods:**

Nasal mucosal tissues of AR patients were collected to determine ACE2 levels. Following AR mouse models were established, ACE2 levels in nasal mucosa were determined. Then the influences of diminazene aceturate (ACE2 agonist) on AR symptoms, pathology, specific antibodies, histamine, and interleukins (ILs) release in vivo were evaluated. Afterward, human nasal mucosa epithelial cells were exposed to IL‐13, and the impacts of ACE2 overexpression on the secretion of pro‐inflammatory factors in vitro were assessed.

**Results:**

ACE2 levels significantly declined in nasal mucosa both in patients and mouse models (*p* < .001). Diminazene aceturate treatment elevated the ACE2 level in mice (*p* < .01), accompanied by reduced frequency of nasal spray and nasal friction, decreased eosinophils and goblet cells (*p* < .001) according to histopathological staining. Furthermore, lgE, lgG1, histamine, and IL levels in mice were also decreased (*p* < .05). In vitro experiments revealed that ACE2 overexpression suppressed the secretion of pro‐inflammatory factors (*p* < .001).

**Conclusion:**

Together, ACE2 activation can alleviate the symptoms of AR in mice and inhibit the release of Th2 cytokines. Activating ACE2 is a promising therapeutic approach for AR.

## Graphical abstract

This study found that angiotensin converting enzyme 2 activation can alleviate the symptoms of allergic rhinitis in mice and inhibit the release of Th2 cytokines. Activating angiotensin converting enzyme 2 is a promising therapeutic approach for allergic rhinitis.

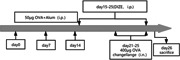

## 1 INTRODUCTION

Allergic rhinitis (AR) is a persistent chronic inflammation of the nasal cavity caused by an aberrant reactivity to some external substances.^1^ These substances that induce aberrant reactions in the nasal passages are called allergens.^2^ Patients usually do not experience an allergic reaction the first time they are exposed to the allergen. Nevertheless, lymphocytes in the body remember the allergen and boost the creation of allergic immunoglobulin E (IgE) from B lymphocytes via the mediator of the cluster of differentiation 4 (CD4) T lymphocytes.^3^ IgE will attach to the surface of mast cells.^4^ When the patient is exposed to the allergen again, the allergen will bind to IgE, triggering the activation of mast cells and release of a substantial amount of histamines, prostaglandins, and leukotrienes, which cause a series of allergic reactions and symptoms.^5^ These reactions enhance the permeability of the blood vessel wall and drive eosinophils to accumulate in the nasal mucosa cells, generating nasal mucosa edema and congestion, resulting in a considerable rise in nasal secretions and a runny nose.^6^ Furthermore, local reactions may excite nerves. Sneezing and nose irritation are classic AR symptoms caused by the peripheral.^7^

The renin‐angiotensin system (Ras) is involved in the immuno‐inflammatory disease process of various tissues and organs.^8^, ^9^ The well‐known Ras signaling pathway is the angiotensin converting enzyme (ACE)‐angiotensin (Ang) II ‐angiotensin II receptors (AT1) axis.^10^ ACE is a zinc metallopeptidase that is generated under the command of the ACE gene. Its major function is to convert Ang I into Ang II, thereby exerting biological effects including vasoconstriction, bradykinin inactivation, and hydrolysis of Ang (1‐7) into inert peptides.^11^ In 2000, the first ACE‐related homologous gene, ACE2, was found and cloned.^10^ ACE2 is a monocarboxypeptidase that metabolizes various peptides, including the degradation of Ang II to Ang 1‐7, and exerts vasodilator/antiproliferative effects through the Mas receptor.^12^ Interestingly, ACE2 is the receptor for SARS‐CoV‐2, and its expression in allergic airway disease may lessen the risk and severity of COVID‐19.^13^ It also has a protective effect on allergic symptoms. For example, a study has discovered that activating ACE2 can lower allergic airway inflammation in asthma model rats.^14^ However, its involvement in AR has not been revealed.

Through GSE101720 datasheet data analysis, compared with nasal epithelial cells, ACE2 gene expression was shown to be lower in bronchial epithelial cells of patients with AR and allergic asthma.^15^ Meanwhile, a study indicated that patients with AR had lower levels of ACE2 in their nasal epithelial cells.^16^ Therefore, this study aimed to determine whether ACE2 could participate in the regulation of AR.

## 2 METHODS AND MATERIALS

### 2.1 Clinical samples

Nasal mucosal tissues of AR patients (10 cases) and the same amount of health people as the control group were collected. Inclusion criteria: patients were diagnosed with AR according to ≥2 typical clinical features, positive allergen, and abnormal specific lgE value.^17^ Patient characteristics are listed in Table 1. Exclusion criteria: Patients who have received antihistamine or glucocorticoid treatment, or allergen‐specific immunotherapy in the past month were excluded, and no patients suffered from nasosinusitis, asthma, and other related diseases.

**Table 1:** Baseline information in patients with allergic rhinitis

No.	Gender	Age	Allergen	Symptom: (1) sneezing, (2) rhinorrhea, (3) nasal obstruction, and (4) nasal itching	Specific lgE (kU/L)
1	Female	42	Animal dander	1, 2, 4	53.3
2	Male	29	Pollen	2, 3, 4	22.1
3	Male	32	Pollen	1, 2, 3, 4	19.5
4	Female	47	Dust mites	1, 2, 3	21.3
5	Male	28	Pollen	1, 2, 4	18.7
6	Female	39	Dust mites	1, 2, 3, 4	22.5
7	Female	23	Pollen	2, 4	14.2
8	Male	36	Animal dander	1, 2, 4	21.30
9	Female	37	Pollen	1, 2, 4	48.9
10	Male	45	Animal dander	2, 3, 4	19.6

Abbreviation: lgE, immunoglobulin E.

### 2.2 Animal experiment

**Figure 1:**
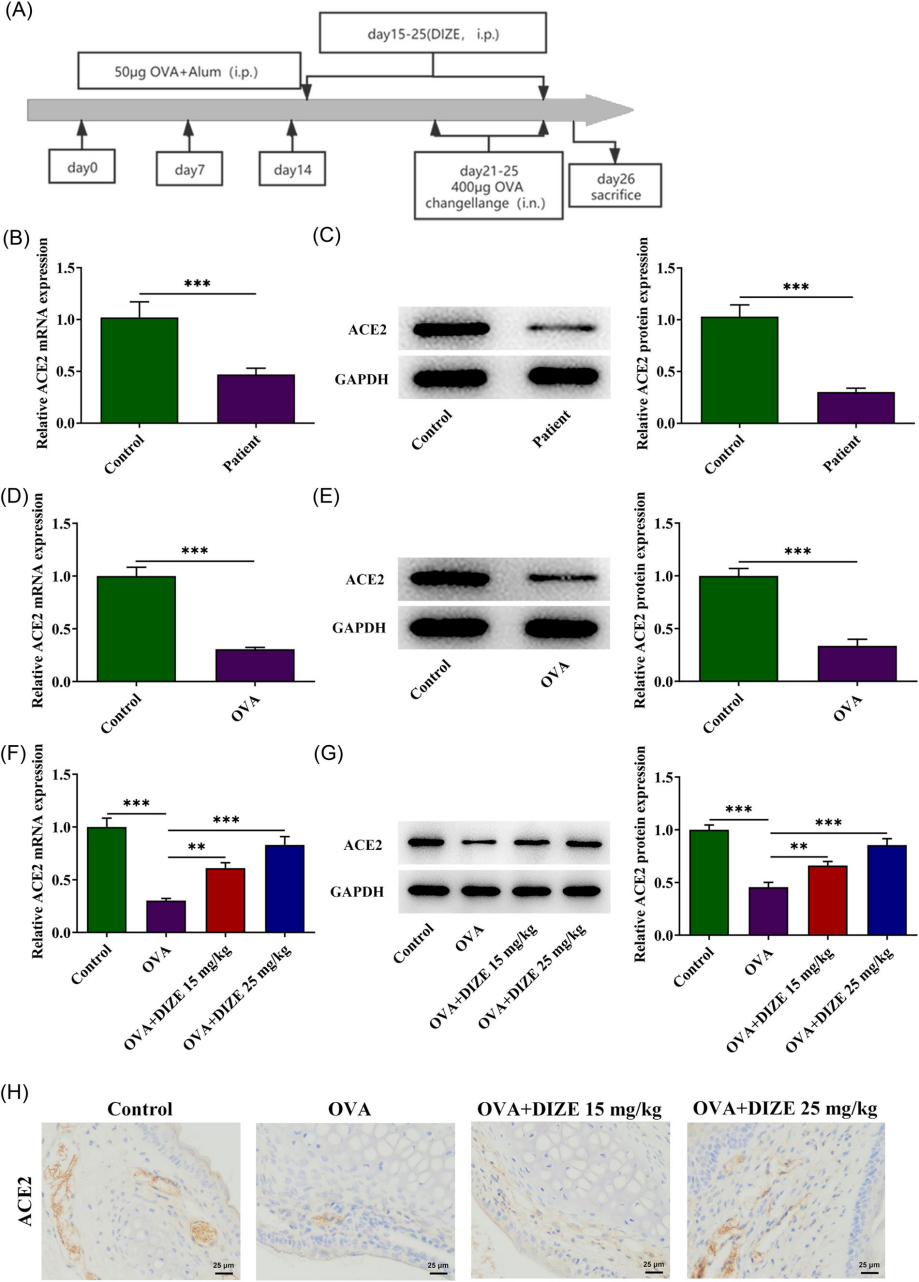
The enrichment of ACE2 in nasal mucosa. (A) The procedure for mice treatment. (B) The enrichment of ACE2 in the nasal mucosa of allergic rhinitis patients was determined by RT‐qPCR and (C) western blot analysis, *n* = 10, unpaired t test. (D) ACE2 levels in the nasal mucosa of allergic rhinitis mice were determined by RT‐qPCR and (E) western blot analysis, *n* = 3, unpaired *t* test. (F) ACE2 levels in the DIZE treatment group were assessed with RT‐qPCR and (G) western blot analysis, *n* = 5, Tukey's test. (H) The enrichment of ACE2 in the nasal mucosa of mice were determined using immunohistochemistry, magnification ×400. *p < .01 and ***p < .001. ACE2, angiotensin converting enzyme 2; DIZE, diminazene aceturate; OVA, ovalbumin; RT‐qPCR, reverse transcriptase‐quantitative polymerase chain reaction.

BALB/c mice (30 male, 20 ± 2 g) were provided by Hunan STA Laboratory Animal Co., Ltd. The procedure was as demonstrated in Figure 1A. Mice in experimental groups were treated with 50 μg ovalbumin (OVA) and aluminum hydroxide mixture on Days 0,  7, and 14 of modeling, and saline was adopted to inject the mice in the control group. Diminazene aceturate (DIZE, 15 or 25 mg/kg), a type of ACE2 agonist, or dexamethasone (Dex, 2.5 mg/kg) ^18^ were applied to administrate mice on Days 15–25 once a day. On Days 21–25, OVA (400 μg) was applied to administrate the mice by nasal. All operations are strictly in accordance with the National Institute of Health Guide for the Care and Use of Laboratory Animals.

### 2.3 **Reverse transcriptase‐quantitative polymerase chain reaction (**RT‐qPCR)

Total RNA from samples was extracted with Trizol reagent, the lysate was centrifuged at 12,000*g* for 12 min at 4°C, and the RNA was precipitated with isopropanol. The RNA was processed into complementary DNA (cDNA) with the cDNA Synthesis Kit, according to the instruction manual. RT‐qPCR was carried out with the SYBR Premix Ex Taq II kit (Takara). A total of 20 µl of the solution constituted the reaction system, including Master Mix, DNA template, and validated specific primers. Thermocycling conditions of the qPCR were: 5 min at 95°C, with 40 cycles for 30 s at 95°C and 45 s at 65°C. The comparative *C*_t_ method was implemented to calculate relative messenger RNA levels and glyceraldehyde‐3‐phosphate dehydrogenase (GAPDH) was used as an endogenous control.

### 2.4 Western blot analysis

Samples were lysed with radio‐immunoprecipitation assay lysis buffer, centrifuged at 12,000*g* for 10 min at 4°C, and the supernatant was collected. Proteins were quantified with a bicinchoninic acid assay kit. Separating gel and stacking gel were prepared separately, and 40 µg protein per well was electrophoresed, followed by repositioning onto polyvinylidene fluoride membranes. The membranes were blocked with 5% skimmed milk at room temperature for 1 h, primary antibodies against ACE2 (1:1000 diluted; Abcam) and GAPDH (1:1000 diluted; Abcam) at 4°C overnight, and HRP‐labeled goat anti‐rabbit secondary antibody (1:5000 diluted; Abcam). Quantity One software was used to analyze the gray level of the protein and GAPDH was used as an internal reference to calculate the relative levels of the specific proteins.

### 2.5 Symptoms

Following intranasal administration of OVA to mice, the number of nasal sneezing and rubes in the mice within 15 min were observed and recorded.

### 2.6 Hematoxylin‐eosin (H&E) staining

Following the mice were decapitated, the face were placed in 4% paraformaldehyde fixation solution overnight, and then parts such as eyeballs, muscles, and frontal bones were removed, and the nasal mucosa tissue was moved into 30% formic acid–formaldehyde mixture for decalcification. Subsequently, the tissues were fixed, dehydrated with graded alcohol, and embedded in paraffin. The wax block was cut into slices with a thickness of 4 µm, and the slices were successively treated with xylene and gradient alcohol. Then pathological changes in nasal mucosa tissues were assessed under a microscope (magnification ×400; Leica) after staining was performed with hematoxylin solution for 5 min and eosin for 2 min at room temperature.

### 2.7 Periodic acid‐Schiff (PAS) staining

Paraffin sections were deparaffinized as abovementioned and immersed in periodate solution for 15 min. The sections were then washed with distilled water and immersed in Schiff's solution for 30 min at room temperature. Nuclei were counterstained with hematoxylin for 3 min. After dehydration with ethanol, permeabilization with xylene, and mounting, the sections were observed under a microscope (magnification ×400).

### 2.8 Enzyme‐linked immunosorbent assay (ELISA)

IgE, lgG1/2, histamine levels in the serum, and Th2 anti‐inflammatory factors (IL‐4, IL‐5, IL‐13) in the serum and nasal lavage were determined using commercial ELISA kits according to the respective kit instructions. Briefly, samples were incubated sequentially with biotinylated antibody, avidin–peroxidase complex, 3,3',5,5'‐tetramethylbenzidine developing solution, and stop solution. The optical density (OD) value was recorded and the results were obtained according to the standard curve.

### 2.9 Immunohistochemistry

Paraffin sections were rehydrated with alcohol, antigen retrieved, and blocked with 3% goat serum for 1 h. Sections were then coincubated with biotin‐labeled secondary antibodies (ZSGB‐BIO) after IL‐4 primary antibody (Abcam) incubation. Proteins were visualized as brown pigments via a standard diaminobenzidine protocol. Afterward, sections were lightly counterstained with hematoxylin and immunostaining was observed under a microscope (magnification ×400).

### 2.10 Cell culture and treatment

Human nasal mucosa epithelial cells HNEpC (BNCC356247; BeNa Culture Collection) were maintained in Eagle's minimum essential medium with 10% fetal bovine serum (Gibco) at 37°C in the presence of 5% CO_2_. Cells were transfected with plasmids to overexpress ACE2 using X‐tremeGENE HP transfection reagent (Roche). The transfection reagent and plasmids were mixed gently for 15 min before being added to the cells. Following 48 h, cells were treated with IL‐13 (10 ng/ml) for 48 h. Untreated cells were regarded as the control.

### 2.11 Statistical analysis

All data displayed as mean ± SD got analyzed with Prisma 7.0 software. To demonstrate comparisons among multiple groups, one‐way analysis of variance was utilized, followed by Tukey's test. For two groups, unpaired *t* test was applied. *p* < .05 is statistically significant.

## 3 RESULTS

### 3.1 The enrichment of ACE2 in the nasal mucosa

The enrichment of ACE2 in the nasal mucosa of AR patients were determined by RT‐qPCR (Figure 1B) and western blot analysis (Figure 1C). Compared with the control, ACE2 levels significantly declined in the patients. Thereafter, following the decreased levels of ACE2 in the nasal mucosa of AR mice were also confirmed (Figure 1D,E), ACE2 levels in the DIZE treatment group were assessed using RT‐qPCR (Figure 1F), western blot analysis (Figure 1G), and immunohistochemistry (Figure 1H). DIZE treatment elevated the ACE2 level in the mice, which alleviated the stimulation of OVA.

### 3.2 Effects of ACE2 activation on symptoms and pathology

**Figure 2:**
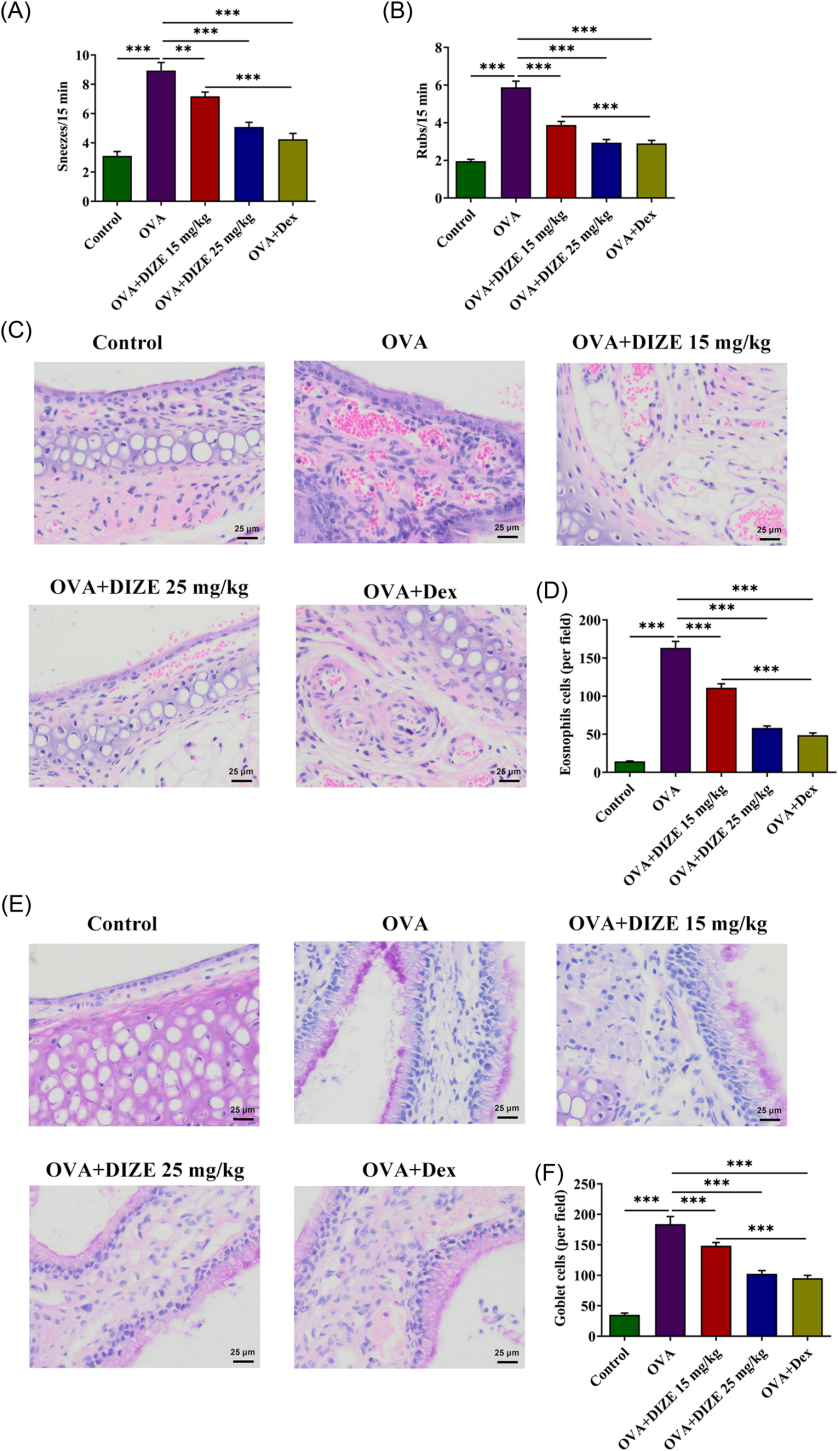
Effects of ACE2 activation on symptoms and pathology (A) The number of nasal sprays and (B) nasal friction in model mice treated with DIZE and Dex and control mice within 15 min were recorded. (C, D) Nasal mucosa tissue of mice was subjected to H&E staining, magnification ×400. (E, F) Mucosal goblet cells were observed by PAS staining, magnification 400×. *n* = 5, Tukey's test, **p < .01 and ***p < .001. ACE2, angiotensin converting enzyme 2; DIZE, diminazene aceturate; H&E, hematoxylin‐eosin; OVA, ovalbumin; PAS, periodic acid‐Schiff.

In subsequent experiments, Dex treatment was used as a positive control. The number of sneeze (Figure 2A) and nasal friction (Figure 2B) in model mice treated with DIZE and Dex and control mice within 15 min were recorded, respectively. Compared with the control group, the number of nasal sprays and nasal friction of the model mice was significantly increased. DIZE treatment significantly reduced the number of times, and with increasing concentrations, the number of times was less. The number of times in the DEX control group was also significantly lower than that in the model group. According to the H&E staining results (Figure 2C,D) of nasal mucosa tissue of mice in each group, there was obvious inflammatory infiltration in the model group, and eosinophils were significantly increased. Whereas this phenomenon was markedly alleviated in the treated mice. Moreover, the PAS staining results (Figure 2E,F) indicated that mucosal goblet cells proliferated obviously in the model group. Similarly, this phenomenon was alleviated in the treated mice.

### 3.3 Effects of ACE2 activation on release

**Figure 3:**
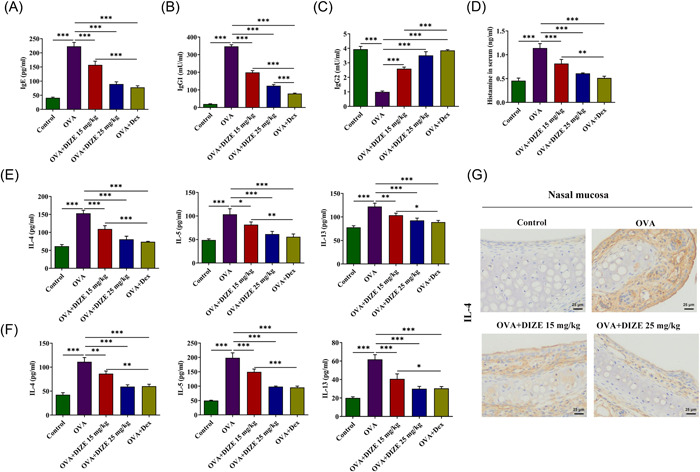
Effects of ACE2 activation on release. (A) The levels of lgE, (B) lgG1, (C) lgG2, and (D) histamine in the serum of mice were determined using ELISA assays. (E) The levels of IL‐4, IL‐5, and IL‐13 in nasal lavage fluid and (F) serum were measured using ELISA assays. (G) IL‐4 enrichment in the nasal mucosa was demonstrated by immunohistochemistry, magnification 400×. *n* = 5, Tukey's test, *p < .05; **p < .01 and ***p < .001. ACE2, angiotensin converting enzyme 2; DIZE, diminazene aceturate; ELISA, enzyme‐linked immunosorbent assay; H&E, hematoxylin‐eosin; lgE, immunoglobulin E; IL, interleukin; OVA, ovalbumin; PAS, periodic acid‐Schiff.

Afterward, the levels of lgE (Figure 3A), lgG1 (Figure 3B), lgG2 (Figure 3C), and histamine (Figure 3D) in the serum of mice were determined using an ELISA assay. Compared with the control group, the IgE level in the model group increased nearly five times, and the IgE levels in the DIZE and Dex treatment groups were significantly lower than those in the model group. High concentrations of DIZE caused similar declines as Dex, but both were still higher than those in the control group. As for the IgG1 level, the model group was greatly improved compared with the control group. The IgG1 levels of the DIZE and Dex treatment groups were significantly lower than those of the model group, and the decreased effect caused by Dex treatment was better. For IgG2 levels, the model group was lower than the control group, and the levels in both DIZE and Dex‐treated groups were significantly higher than those in the model group. The histamine level in the model group was higher than that in the control group, which was decreased by the additional DIZE and Dex treatment.

The levels of IL‐4, IL‐5, and IL‐13 (Figure 3E) in nasal lavage fluid were measured using ELISA assays. The levels of these three factors were significantly increased in the model group, and DIZE and Dex treatment could effectively reduce them. In addition, the levels of these three factors in serum were also detected to be reduced by DIZE and Dex treatment (Figure 3F). Furthermore, IL‐4 enrichment in the nasal mucosa was demonstrated by immunohistochemistry (Figure 3G). The fluctuation trends of IL‐4 in each group was consistent with those in nasal lavage fluid and serum.

### 3.4 ACE2 overexpression suppresses inflammatory release in vitro

**Figure 4:**
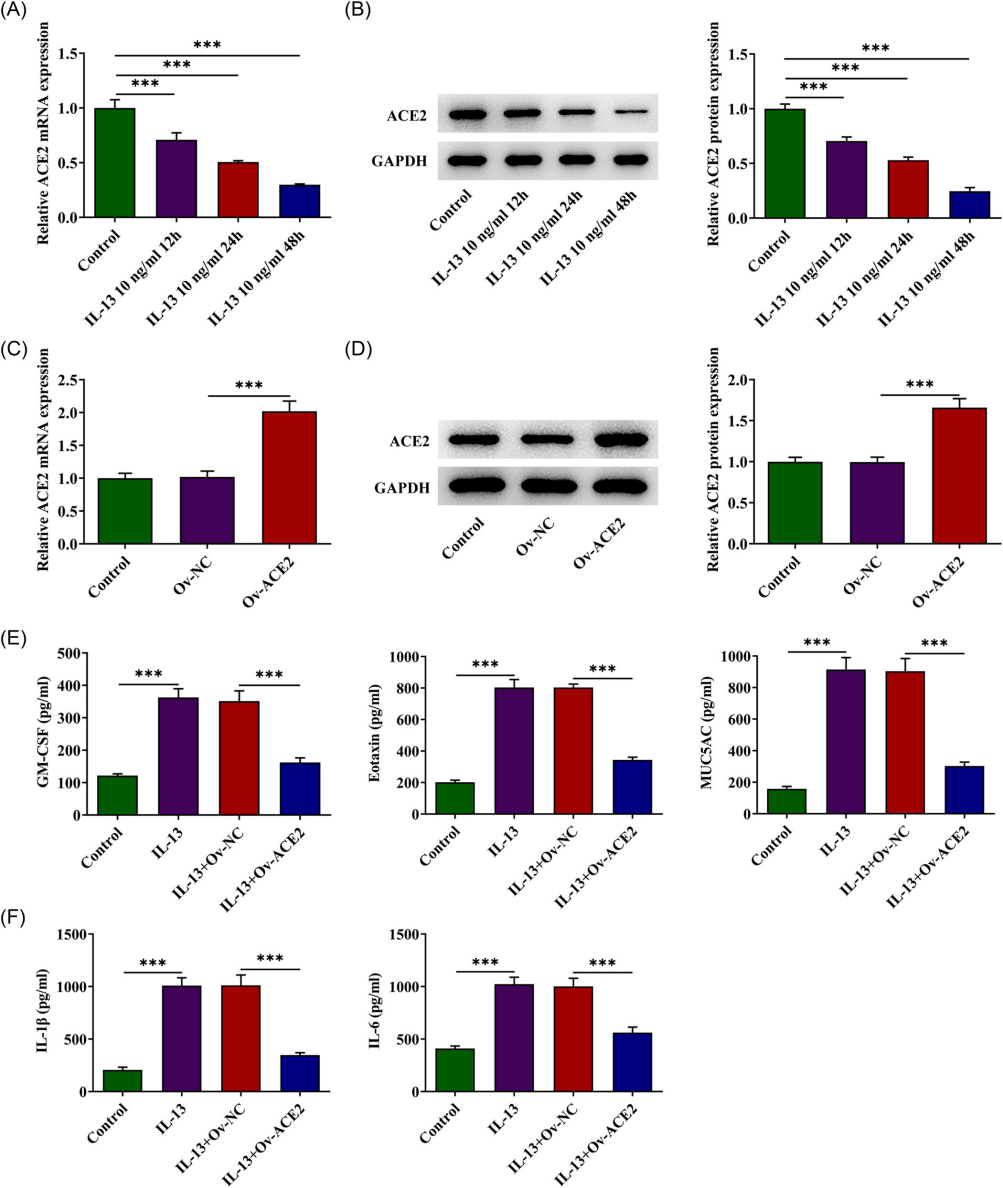
ACE2 overexpression suppresses inflammatory release in vitro. (A) ACE2 levels were determined in HNEpC exposed to IL‐13 for 12, 24, and 48 h using RT‐qPCR and (B) western blot analysis. (C) ACE2 overexpression in HNEpC was confirmed using RT‐qPCR and (D) western blot analysis. (E) The levels of GM‐CSF, Eotaxin, and MUC5AC were detected by ELISA assay. (F) IL‐1β and IL‐6 levels were examined using ELISA assays. *n* = 3, Tukey's test, ***p < .001. ELISA, enzyme‐linked immunosorbent assay; GM‐CSF, granulocyte‐macrophage colony‐stimulating factor; IL, interleukin; RT‐qPCR, reverse transcriptase‐quantitative polymerase chain reaction.

ACE2 levels were determined in HNEpC exposed to IL‐13 for 12, 24, and 48 h using RT‐qPCR (Figure 4A) and western blot analysis (Figure 4B). The level of ACE2 in the cells continued to decrease with increasing treatment time. Then ACE2 was forced to overexpress in cells (Figure 4C,D), and the levels of granulocyte‐macrophage colony‐stimulating factor (GM‐CSF), eotaxin, and MUC5AC (Figure 4E) were detected by ELISA assays. All three inflammatory cytokines, especially Eotaxin and MUC5AC, were greatly elevated after IL‐13 treatment, whereas ACE2 overexpression alleviated their elevated levels in cells. In addition, IL‐1β and IL‐6 (Figure 4F) levels were also examined, which were also reduced by ACE2 overexpression, reversing the effects of IL‐13 treatment.

## 4 DISCUSSION

AR is primarily regulated by type I hypersensitivity,^19^ with Th2 and IgE playing essential roles. When an allergen enters the body, it is first presented to the immune system by antigen‐presenting cells such as dendritic cells, and then directed by a Th2 mediator, triggering a series of related interleukin effects, and finally causing plasma B cells to produce corresponding IgE to deal with invading allergens.^20^ Th2 has a very significant influence on persons with allergic constitutions, and it induces more IgE than normal people. As a response, physicians will provide customized treatment based on particular IgE detection.^21^ Importantly, the current study found that ACE2 agonist therapy decreased the release of IgG in vivo in mice, which was accompanied by a decrease in IgG1, histamine, and interleukin. The symptoms of AR in mice were likewise alleviated during this process.

The nasal mucosa is the first line of defense against environmental allergens,^22^ and in vitro models using nasal mucosal epithelial cells exhibit similar responses to exogenous stimuli, rendering them important for identifying therapeutic targets for AR patients.^23^ Mucous cell metaplasia, epithelial fibrosis, smooth muscle hypertrophy, and other pathological processes in allergic disorders have been associated with IL‐13, a Th2 cytokine.^24^, ^25^, ^26^ The IL‐13 signaling pathway is important in the pathogenesis of AR by increasing the release of inflammatory cytokines (such as eotaxin, GM‐CSF, and IL‐1)^27^ and stimulating mucus production in nasal epithelial cells of AR patients.^28^ Therefore, this study continued to investigate whether ACE2 could affect IL‐13‐induced inflammatory cytokine release in nasal mucosal epithelial cells. The findings of the experiments revealed that ACE2 overexpression partially resisted the stimulation of IL‐13 and attenuated the inflammatory response. Because of the global outbreak of COVID‐19, ACE2‐related research is primarily focused on COVID‐19.^29^, ^30^ SARS‐CoV2 enters cells via membrane fusion and dramatically downregulates the ACE2 receptor, resulting in the loss of its catalytic function at the membrane's periphery. Following the viral invasion, additional ACE2 deficit may amplify dysregulation of the ACE2 regulatory axis.^31^ Nevertheless, this study is limited to the focus on ACE2, and its upstream and downstream are still unknown. Our team will continue to study the involvement of the ACE2 regulatory axis in AR.

## 5 CONCLUSION

Together, this study found that ACE2 activation can alleviate the symptoms of OVA‐induced AR in mice and inhibit the release of Th2 cytokines. Activating ACE2 is a promising therapeutic approach for AR.

## AUTHOR CONTRIBUTIONS

Xiuying Sun and Yu Xu contributed to the concept, experiments, and draft, and Jinhui Zhou contributed to the experiments and data analysis. Xiuying Sun, Yu Xu, and Jinhui Zhou approved the final version.

## CONFLICT OF INTEREST

The authors declare no conflict of interest.

## ETHICS STATEMENT

This study was approved by The Affiliated Huaian No. 1 People's Hospital of Nanjing Medical University (KY‐2022‐027‐01). All the patients provided informed consent upon this study.

## Data Availability

The datasets utilized and/or analyzed in this study are available from the corresponding author on reasonable request.
